# Continuous activation of the IL-17F driven inflammatory pathway in acute and chronic digital dermatitis lesions in dairy cattle

**DOI:** 10.1038/s41598-022-17111-4

**Published:** 2022-08-18

**Authors:** Anne-Sofie Vermeersch, Peter Geldhof, Richard Ducatelle, Yannick Gansemans, Filip Van Nieuwerburgh, Dieter Deforce, Geert Opsomer

**Affiliations:** 1 Department of Internal Medicine, Reproduction and Population Medicine, Faculty of Veterinary Medicine, Ghent University, 9820 Merelbeke, Belgium; 2 Department of Translational Physiology, Infectiology and Public Health, Faculty of Veterinary Medicine, Ghent University, 9820 Merelbeke, Belgium; 3 Department of Pathobiology, Pharmacology and Zoological Medicine, Faculty of Veterinary Medicine, Ghent University, 9820 Merelbeke, Belgium; 4 Laboratory of Pharmaceutical Biotechnology, Faculty of Pharmaceutical Sciences, Ghent University, 9000 Ghent, Belgium

**Keywords:** Inflammation, Skin diseases, Gene expression

## Abstract

Objectives of the present study were to get a deeper insight into the course of the inflammatory pathways of digital dermatitis lesions in dairy cattle by investigating the gene expression patterns throughout the different clinical stages (M0 to M4.1) of the disease. Normal skin samples (M0) were used as a reference for comparing the gene expression levels in the other M-stages through RNA Seq-technology. Principal component analysis revealed a distinct gene expression pattern associated with digital dermatitis lesions in comparison to healthy skin with a further clustering of the acute M1, M2 and M4.1 stages versus the chronic M3 and M4 stages. The majority of the up-and downregulated genes in the acute and chronic stages can be placed into a common ‘core’ set of genes involved in inflammation, such as A2ML1, PI3, CCL11 and elafin-like protein, whereas the most downregulated genes included keratins and anti-inflammatory molecules such as SCGB1D and MGC151921. Pathway analysis indicated the activation of the pro-inflammatory IL-17 signaling pathway in all the M stages through the upregulation of IL-17F. These results indicate that digital dermatitis is associated with an excessive inflammatory immune response concomitant with a disrupted skin barrier and impaired wound repair mechanism. Importantly, despite their macroscopically healed appearance, a significant inflammatory response (Padj < 0.05) was still measurable in the M3 and M4 lesions, potentially explaining the frequent re-activation of such lesions.

## Introduction

Foot and leg problems constitute major health and welfare issues and entail substantial economic losses in modern dairy farming^1^. These losses are due to the treatment costs and costs associated with the reduction of milk yield and reproductive performance^1,2^. The most commonly occurring skin condition of the distal limb in cattle is digital dermatitis (DD), also known as Mortellaro’s disease, usually located on the plantar surface of the hind feet. Based on the clinical appearance, a macroscopic classification of six stages (M0–M4.1) was introduced by Döpfer et al. and Berry et al.^3,4^. The first M-stages M1 and M2 represent ‘acute’ lesions characterized by painful ulcerations. These lesions gradually transform to the M3 stage with the formation of a dry crust on the surface of the lesion and subsequently to the M4 lesion which represents a macroscopically healed lesion. Finally, the category ‘M4.1’ is characterized by an apparent re-activation of the lesion, macroscopically resembling an M1-stage lesion. At the moment, footbathing and topical treatment have been proven to be valuable but are not efficient to eradicate the disease, not at cow nor at herd level^3,5^.

This dynamic disease is considered to be multifactorial and polymicrobial, with multiple species of the anaerobic *Treponema* genus as the key players^6,7^. The characteristic lesions tend to be quite painful hence causing severe discomfort and lameness. The tissue destruction is likely mediated by a dysregulated inflammatory response of the host as well as by presumable virulence factors of involved bacteria, leading to the typical macroscopic presentation.

Research into the pathogenesis of the disease has shown that lesions are characterized by hyperplasia and hyperkeratosis of the epidermis, elongation of the rete ridges and desquamation due to the inflammatory response^4,8^. Lesion development is further associated with the infiltration of neutrophils and eosinophils^4,8^ as well as a dramatic increase in keratinocyte-derived interleukin-8 (IL-8) levels^8–10^. Furthermore, several gene networks involved in skin matrix turnover and inflammation have been shown to be upregulated in the acute M2 stage in comparison to healthy skin, including various chemokines, A2-macroglobuline-like 1 and matrix metalloproteinases^9^. In contrast, several keratins and keratin-associated proteins appear to be significantly downregulated (Padj < 0.05)^9^. Importantly, despite the induction of an adaptive immune response, as reflected by the generation of anti-treponeme antibodies^11^, animals apparently do not develop protective immunity and remain susceptible for the clinical re-emergence of DD lesions.

Most of our knowledge on the pathogenesis of the disease comes from the acute M2 lesions. Information on the processes involved in the progression of the lesions from the acute M1 and M2 stages towards the chronic ‘dormant’ stages, and their potential reactivation, is largely missing. To address this, the aim of the current study was to examine and compare changes in gene expression levels in all clinical M-stages by RNA deep sequencing analysis.

## Materials and methods

### Sample collection and handling

The applied sampling protocol was approved by the ethical committee of the Faculty of Veterinary Medicine of Ghent University (dossier number 2018-13). Samples were taken in accordance with the relevant guidelines and regulations and all authors complied with the ARRIVE guidelines. Most samples were taken in average sized dairy farms with identical husbandry practices such as limited pasture access, presence of concrete slatted floors with cubicles and milking by a robotic milking system. In total, 22 skin samples were obtained from 5 different M stages (M1, M2, M3, M4, M4.1) on 6 dairy farms with Holstein–Friesian cows. The sampled population consisted of adult, lactating cows of a variable age. Five M0 samples and two M1 samples were harvested from Holstein–Friesian cattle postmortem at the local slaughterhouse (Zele, Belgium), after they were clinically scored for absence of lameness prior to slaughter. Feet were defined as 'healthy' (M0) when having a macroscopically healthy skin with the absence of any kind of lesion. Cows with concurrent non-DD related feet and leg disorders, or in the case of the samples of live animals, showing lameness, were excluded from this study. Lesions were categorized from M1 to M4.1 according to the M-stage scoring system set up by Döpfer et al. and Berry et al.^3,4^. Lesions proximal to the interdigital cleft were taken and sampling was restricted to only 1 lesion site per cow. The samples were taken with a sterile, disposable 4 mm biopsy punch tool (Kai Medical, Solingen, Germany) and immediately put in a liquid nitrogen container (− 196 °C). Following transport to the research facility, the samples were stored in a − 80 °C freezer until further processing. A second sample was taken with a 4 mm biopsy punch tool, put in 10% neutral buffered formalin and brought to the histology lab for further processing. Post-biopsy, oxytetracycline spray was administered on the lesions before applying a gauze and a self-adhering bandage. Within the following week, the bandage was removed by the farmers.

For isolating RNA, the skin samples were put in a styrofoam container with liquid nitrogen. RNA isolation was performed using the Qiagen RNeasy Minikit (Qiagen Benelux, Venlo, The Netherlands) in an RNase-free environment. The RNA quantity and quality were evaluated with the Experion automated electrophoresis station, utilizing the Experion™ RNA StdSens kit (Bio-Rad, Temse, Belgium). Samples of the digital dermatitis lesions had a mean RQI of 9.5 (standard deviation 0.67) and in healthy M0 samples, the mean RQI was 8.8 (standard deviation 0.82). The RNA was stored in a − 80 °C freezer until shipment to NxtGent (Ghent University, Ghent, Belgium) for Illumina sequencing.

### Histological examination

A hematoxylin–eosin staining was applied to sections obtained from the samples that were fixated in 10% neutral buffered formalin, and subsequently dehydrated and embedded in paraffin. The staining was applied to paraffin embedded tissue sections of 5 µm thick with the Shandon Varistain Gemini autostainer (Thermo Fisher Scientific, Geel, Belgium). The HE staining was used to observe the characteristics of the skin tissue (thickness epidermis, cell infiltration and rete ridges) in order to microscopically confirm digital dermatitis. The slides were mounted for examination with light microscopy on a Leica DM LB2 microscope (Leica Microsystems, Diegem, Belgium).

### RNA sequencing

Sequencing libraries were constructed using the ‘QuantSeq 3’ mRNA-Seq Library Prep Kit FWD for Illumina’ from Lexogen (Lexogen Inc, Greenland, NH, USA) and were sequenced as single-end 75 on an Illumina NextSeq (Illumina Inc, San Diego, CA, USA) using 4 lanes. For each sample, the reads from different lanes were pooled. Quality and length of raw sequencing reads were inspected with FastQc (v0.11.7)^12^. Fastq_screen (v0.11.4) was utilized for checking putative contamination^12^. Adaptor and quality trimming was done using cutadapt (v2.8)^13^. Reads containing ambiguities or not passing the phred score threshold of 20 were removed. The remaining reads were inspected with FastQc (v0.11.7).

### Differential gene expression analysis

The reads were mapped on the Bos_taurus_ARS-UCD1.2 cow reference genome using splice-aware Spliced Transcripts Alignment to a Reference (STAR) (v2.5.3a) mapper and features were counted at the gene and transcript isoform level using rsem_calc_expression (RSEM v1.3.1)^14,15^. To inspect the samples for outliers or labelling errors, PCA plots were generated in R after transforming the raw gene counts with rlog() of the DESeq2 package to stabilize variances. Differential gene expression analysis was done by pairwise comparisons between gene-level expression counts of M0 and each of the different disease stages, using the edgeR package in R^16^. Briefly, lowly expressed features with counts less than 1 count per million in more than the number of samples in the smallest group were removed. Then, counts were normalized using the TMM method and dispersion was calculated before applying the quasi-likelihood model and F-test to perform gene-wise statistical tests. The Benjamini–Hochberg method was used for multiple testing correction in order to control the false discovery rate^17^. The following pairwise comparisons were examined: M0 vs M1, M0 vs M2, M0 vs M3, M0 vs M4 and M0 vs M4.1. For each comparison, gene ontology (GO) term enrichment and pathway enrichment analyses were performed in R with the GAGE package^18^ using the gene fold-changes obtained from the differential expression analysis.

### qPCR analysis

To confirm the results of the RNA sequencing, 3 genes were selected for performing qPCR: interleukin-8 (IL-8); skin-derived antileukoprotease (SKALP); alpha-2-macroglobulin-like 1 (A2ML1). The housekeeping genes RPLP0 (60S acidic ribosomal protein P0) and GAPDH (glyceraldehyde-3-phosphate dehydrogenase) were utilized as reference genes for the normalization of the data based on the normalization factors calculated in Genorm^19^. Relative quantities (Q values) were calculated using the delta Ct method to determine the fold changes in gene transcription levels. Mean fold changes in gene transcription levels were obtained by comparing the different M-stages (M1–M4.1) with healthy M0-stage samples. Primers were designed using PrimerBLAST, based on the bovine reference gene sequences from the National Centre for Biotechnology Information (NCBI) database. A BLAST of the primer sequences was done against the Refseq database of Bos taurus. SYBR Green Master Mix (Applied Biosystems, Ghent, Belgium) was used in the amplification reaction, carried out in the StepOnePlus Real-Time PCR System (Applied Biosystems, Ghent, Belgium). A no template control (NTC) was added as a negative control in order to check for primer-dimer formation and contamination. This contained the RT-PCR reagents with the RNA template substituted by nuclease-free water. Characteristics for the primers used in the present study can be found in supplementary file 6. The qPCR data was statistically analyzed with JASP software^20^. The variations in gene expression were detected by utilizing the nonparametric Mann Whitney U test. A p-value of < 0.05 was considered as significant. Data are available on request.

## Results

### Histology

**Figure 1:**
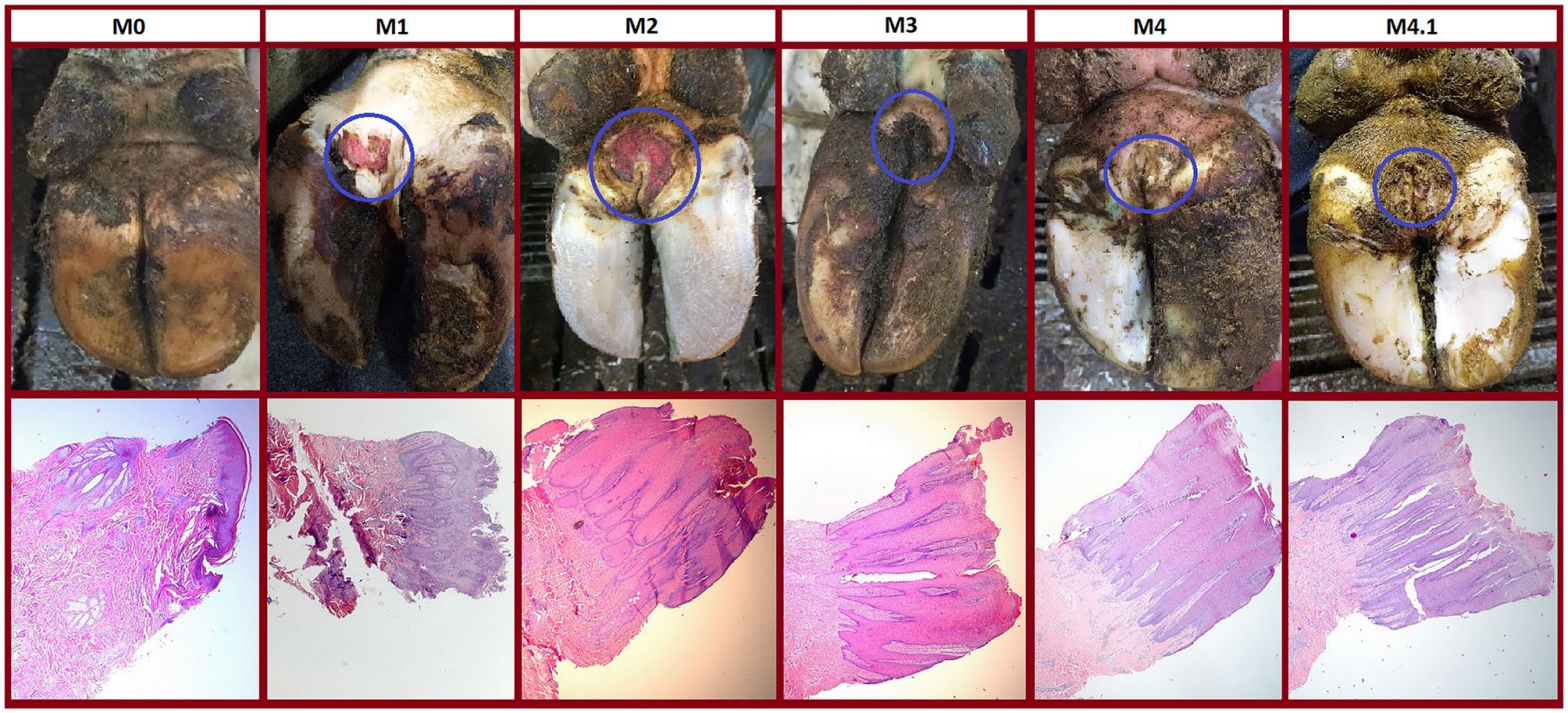
Macro-and microscopical display of all digital dermatitis stages. The top row shows the M-stages, from the healthy skin on the lift to an M4.1 lesion on the right. The lesions are indicated with a circle. The HE stainings of these lesions are displayed in the bottom row (magnification 25×). The separate images have been provided by AV.

The results of the macroscopic and histological analyses are shown in Fig. 1. Our findings were in concordance with findings that have previously been described in literature^3,4,8^. The healthy and the DD affected samples can be distinguished both macroscopically and histologically (Fig. 1). The histological differences within the acute M1 and M2-stages and the chronic M3- and M4-stages are harder to discern. As can be seen in the HE-stainings, the most prominent feature of chronic DD is the massive thickening of the epidermis. In acute lesions, haemorrhages and loss of the epithelium is visible. Moreover, an elongation of the rete ridges and lymphoplasmocytic and neutrophilic infiltration is seen in all stages.

### RNA sequencing and differential gene expression analysis

**Figure 2:**
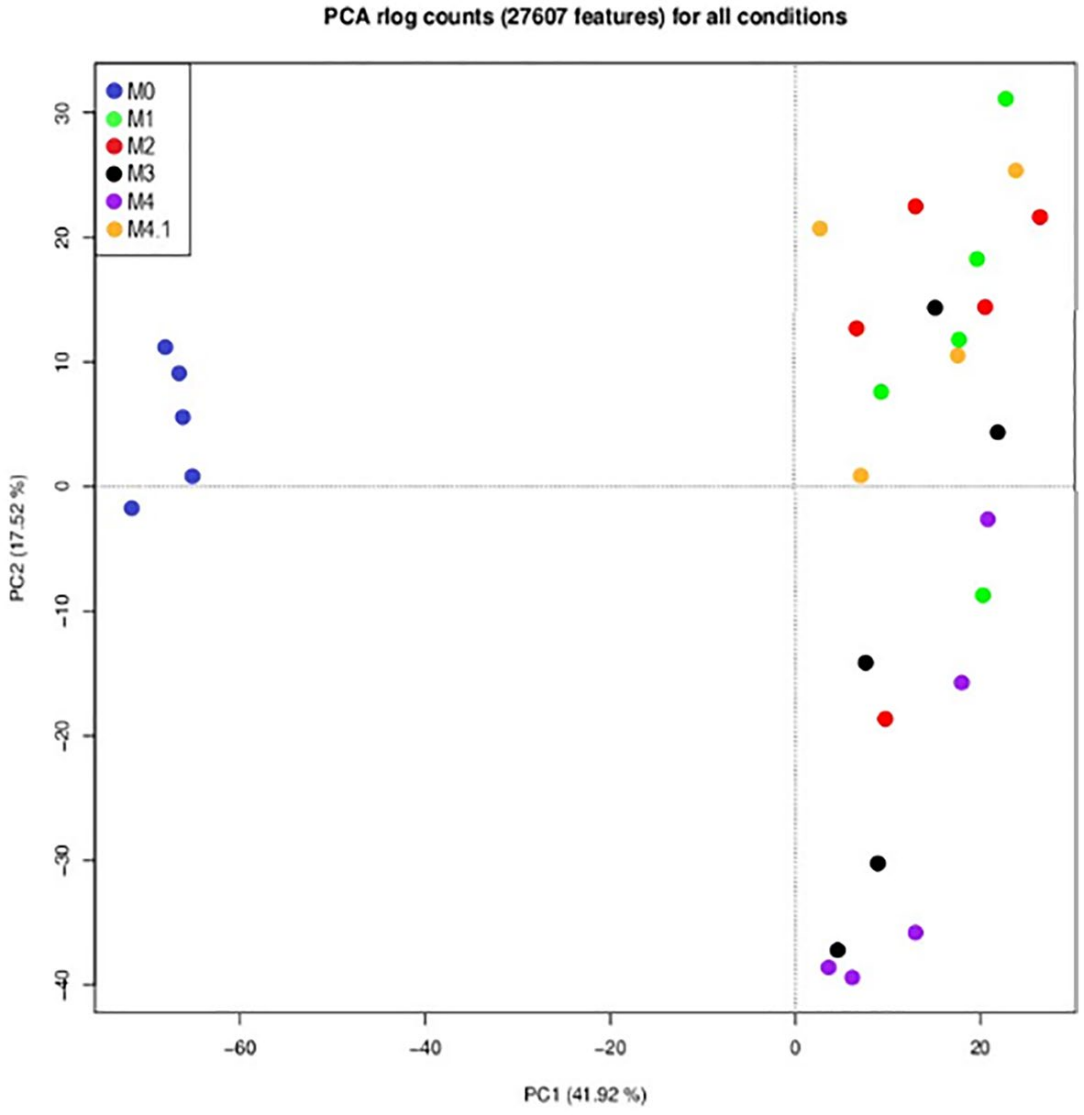
PCA plot of the data. The PCA plot shows all replicates of the different disease stages. Principal components were calculated after variance stabilizing ‘regularized log’ (rlog) transformation of the raw gene counts. Figure made in R version 4.1.2 (https://www.r-project.org) supplied by YG.

Analysis of the generated sequences showed that good quality libraries with an average size of 7.7 million reads (S^2^ = 1.87) were obtained for the different samples (Table 1). In a first phase, a principal component analysis was performed for further quality assessment and exploratory analysis of the generated data. The outcome of this analysis is shown in Fig. 2. The PCA plot indicated that M0 samples clustered separately from the samples of the different disease stages. A further clustering of the acute M1 and M2 stages and the chronic M3 and M4 stages was also observed. The differentially expressed genes were subsequently identified by pairwise comparisons between the M0 samples and each of the different disease stages, i.e. M1, M2, M3, M4 and M4.1. The outcome of this analysis is summarized in Table 2. The 10 most up- and downregulated genes can be found in Table 3 and the entire list of statistically significant DEG (PAdj ≤ 0.05) can be found in the Supplementary file 1. The fold change thresholds for the up-and downregulated genes were set at 2 and − 2, respectively. Throughout all clinical M-stages, the majority of the 10 most upregulated molecules remained largely the same whereas the subset of the ten most downregulated genes appeared to be more diverse (Table 3). Among the most upregulated genes were A2ML1, PI3, CCL11, elafin-like and antimicrobial peptides TAP and LAP. The most downregulated genes comprised of keratins, keratin-associated proteins and molecules such as SCGB1D.

**Table 1:** Total number of trimmed sequences in millions generated with FastQC.

	M0	M1	M2	M3	M4	M4.1
Sample 1	8.9	6.6	9.2	7.5	6.7	5.9
Sample 2	7.8	7.4	9.1	8.3	6.5	10.0
Sample 3	8.4	8.6	7.2	6.9	7.0	8.9
Sample 4	9.5	5.5	7.6	5.1	9.6	7.8
Sample 5	9.0	8.4	5.9	5.3	8.2

**Table 2:** Number of differentially expressed (up-and downregulated) genes in the different M-stages in comparison to healthy skin tissue.

	n genes upregulated (Padj ≤ 0.05; FC ≥ 2)	n genes downregulated (Padj ≤ 0.05; FC ≤ − 2)
M1	1059	1380
M2	1041	1345
M3	917	1148
M4	978	1423
M4.1	1063	1398

The fold change (FC) and adjusted p-value (Padj) threshold are shown between brackets. The fold change threshold is set at ≥ 2 for upregulated genes and ≤ − 2 for downregulated genes.

**Table 3:** The 10 most up- and downregulated differentially expressed genes in samples of M1, M2, M3, M4 and M4.1 lesions in comparison to healthy skin tissue.

Acute stages		Chronic stages		
M1	M2	M3	M4	M4.1
PI3 (2111)	SLPI (2297)	PI3 (2250)	A2ML1 (1963)	A2ML1 (1200)
A2ML1 (1516)	PI3 (1234)	A2ML1 (1981)	PI3 (1908)	PI3 (1149)
TAP (570)	A2ML1 (1093)	APOBEC3Z1 (1074)	TAP (1158)	APOBEC3Z1 (817)
IL6 (550)	CCL11 (761)	TAP (824)	APOBEC3Z1 (1116)	CCL11 (657)
CCL11 (529)	APOBEC3Z1 (491)	LAP (629)	LAP (924)	IL6 (559)
TCN1 (523)	elafin-like (470)	TCN1 (519)	Arg1 (630)	CXCL8 (522)
CXCL8 (384)	CXCL8 (383)	CCL11 (340)	elafin-like (604)	elafin-like (516)
elafin-like (299)	IL6 (373)	Arg1 (336)	TCN1 (553)	CXCL2 (476)
LAP (235)	CXCL2 (328)	SLPI (326)	KRT6B (508)	LYZL1 (364)
LYZL1 (224)	TAP (295)	elafin-like (323)	AQP5 (373)	GRO1 (315)
SCGB1D (− 2014)	BDA20 (− 16,979)	KRT31 (− 11,364)	KRTAP4-7 (− 6448)	major allergen I polypeptide chain 1-like (− 3800)
CYP2B6 (− 1852)	MGC151921 (− 7274)	keratin associated protein-like (− 3066)	KRT35 (− 5170)	MUCL1 (− 3641)
major allergen Equ c-1-like (− 1355)	major allergen I polypeptide chain 1-like (− 3752)	GPRC5D (− 2100)	SEC14L6 (− 3576)	BDA20 (− 2861)
KRTAP12-2 (− 1328)	CYP2B6 (− 1947)	KRTAP4-7 (− 2067)	MUCL1 (− 3165)	major allergen Equ c-1 (− 2358)
major allergen I polypeptide chain 1-like (− 1684)	allergen Bos d 2 (− 1933)	FABP9 (− 1753)	keratin associated protein-like (− 3067)	MGC151921 (− 2275)
MUCL1 (− 1389)	ENSBTAG00000050955 (− 1879)	ENSBTAG00000050955 (− 1666)	ELOVL3 (− 2407)	ENSBTAG00000050955 (− 1905)
ENSBTAG00000054258 (− 1154)	prolactin-inducible protein homolog (− 1852)	BDA20 (− 1542)	KRT25 (− 2244)	SCGB1D (− 1140)
KRT89 (− 941)	major allergen Equ c 1-like (− 1424)	prolactin-inducible protein homolog (− 1411)	KRT31 (− 2169)	HSD17B13 (− 967)
prolactin-inducible protein homolog (− 965)	MUCL1 (− 1288)	major allergen Equ c 1-like (− 1264)	KRTAP1-1 (− 1950)	GLYATL2 (− 951)
steroid 17-alpha-hydroxylase/17,20 lyase (− 910)	SCGB1D (− 970)	KRT26 (− 1205)	uncharacterized LOC524771 (− 1798)	prolactin-inducible protein homolog (− 863)

The fold change is shown between brackets behind the gene designation (adjusted p-value < 0.05). Unknown proteins are noted with their Ensembl identity. The fold change threshold is set at ≥ 2 for upregulated genes and ≤ -2 for downregulated genes.

In order to further functionally profile the differentially expressed genes, a gene ontology enrichment analysis was performed. Statistically overrepresented GO terms could be found in the M1 and M2 stages (Supplementary file 2). No GO terms were under-represented. With regards to the M3, M4 and M4.1 stages, no GO terms were statistically significantly over- or under-represented (Padj < 0.05). Additionally, a pathway analysis was performed to identify gene networks impacted in the different disease stages. The pro-inflammatory IL-17 signaling pathway was significantly upregulated in all stages (Padj < 0.05). Genes involved in this pathway that were differentially expressed in the current analysis, are listed in Table 4. Although statistically the IL-17 signaling pathway was not activated in the M3 stage, 31 genes belonging to this pathway, such as IL17F and CCL11, were still upregulated in the M3 stage (Table 4). Next to the IL-17 signaling pathway, an additional 6 pathways were significantly upregulated (Padj < 0.05) in the M2-stage, including pathways linked to cellular proliferation and inflammatory responses (Supplementary file 3). Apart from the upregulated pathways, only one pathway was significantly downregulated (Padj < 0.05), i.e. the steroid hormone biosynthesis pathway in the M1 stage. The differentially expressed genes involved in all these pathways are listed in Supplementary file 3.

**Table 4:** Up-and downregulated genes in the IL-17 signaling pathway for all relevant M-stages.

M1	M2	M3	M4	M4.1
CCL11 (529) CXCL8 (384) CXCL2 (204) CXCL1 (163) CXCL5 (163) COX2 (96) MMP1 (77) MMP9 (70) IL1B (39) MMP3 (37) S100A8 (14) S100A9 (12) FOSL1 (12) GCSF (12) CCL20 (12) TRAF5 (11) IL17F (9) FOS (8) CCL2 (6) MAPK6 (4) MAPK14 (4) TNF (3) Casp 3 (3) A20 (3) HSP90B1 (2) TRAF3 (2) MAPK1 (2) TRAF3IP2 (2) TRADD (2) Casp 8 (2) HSP90AA1 (2) TAK1 (2) SF2 (2) HUR (2)	CXCL8 (383) IL6 (373) CXCL2 (328) CXCL1 (207) PTGS2 (174) CXCL5 (159) IL1B (31) TRAF5 (14) FOSL1 (11) S100A8 (11) S100A9 (10) TNF (8) GCSF (8) CCL20 (7) CXCL3 (7) CCL2 (5) FOS (5) MAPK6 (5) Casp 3 (4) MAPK14 (3) TRAF3 (3) MAP3K7 (2) TRAF3IP2 (2) HSP90B1 (2) MAPK1 (2) SF2 (2) Casp 8 (2) FADD (2) TRADD (2)	CCL11 (341) CXCL8 (299) CXCL2 (258) CXCL1 (149) PTGS2 (74) MMP9 (67) CCL20 (31) MMP1 (31) IL1B (25) S100A9 (21) S100A8 (19) MMP3 (11) TRAF5 (9) FOSL1 (7) FOS (7) IL17F (6) TNF (6) MAPK6 (4) MAPK14 (4) Casp 3 (3) A20 (2) MAPK13 (2) TRAF3IP2 (2) MAPK1 (2) MAP3K7 (2) TRAF3 (2) MAPK3 (2) HSP90B1 (2) TRAF4 (2) SRSF1 (2)	CCL11 (286) CXCL2 (148) CXCL8 (92) MMP9 (38) COX2 (38) S100A9 (24) S100A8 (22) IL1B (13) CCL20 (12) TRAF5 (10) FOSL1 (6) MMP3 (5) FOS (5) MAPL14 (5) CXCL3 (4) MAPK6 (4) MAPK13 (3) MAPK1 (3) TNFAIP3 (2) TRAF3IP2 (2) MAPK3 (2) FADD (2) Casp 3 (2) MAP3K7 (2) TRAF4 (2) TRAF3 (2)	CCL11 (657) CXCL8 (522) CXCL2 (476) COX2 (236) CXCL5 (208) MMP9 (106) MMP1 (93) IL1B (54) MMP3 (32) GCSF (15) S100A8 (11) TRAF5 (11) CXCL3 (9) S100A9 (8) IL17F (8) CCL20 (8) FOS (7) TNF (6) CCL2 (6) MAPK14 (4) MAPK6 (4) TNFAIP3 (3) FOSB (3) TRAF3 (2) HSP90B1 (2) MAPK1 (2) TRAF3IP2 (2) MAPK3 (2) SF1 (2) MAP3K7 (2) TRADD (2)
S100A7 (-112) LCN2 (-9) ANAPC5 (-2)	LCN2 (-11) ANAPC5 (-2)	LCN2 (-7)	LCN2 (-13) TAB2 (-3)	S100A7 (-75) LCN2 (-10) TAB2 (-4)

The fold change is shown between brackets behind the gene designation. The adjusted p-value is < 0.05. The fold change threshold is set at ≥ 2 for upregulated genes and ≤ -2 for downregulated genes.

**Figure 3:**
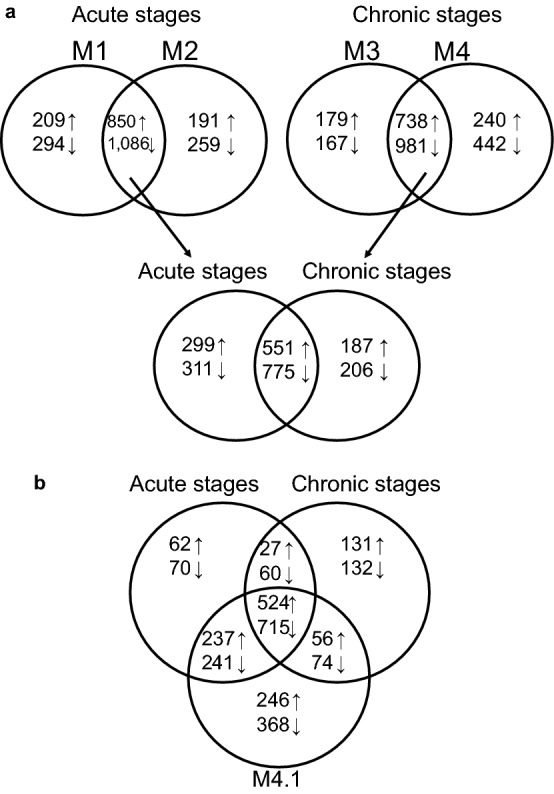
Visualization of the overlap and the unique subsets of the up-and downregulated genes throughout all DD stages. (**a**) the overlap and unique subsets of the up-and downregulated genes in acute (M1, M2) and chronic (M3, M4) stages. (**b**) the overlap and the unique subsets of the up-and downregulated genes throughout the acute (M1, M2), chronic (M3, M4) and M4.1 stages.

The DEGs unique to either the acute or chronic stages were subsequently identified by comparing the overlapping DEGs for both the acute (M1 and M2) and chronic stages (M3 and M4) (Fig. 3a and Supplementary file 4). This resulted in the identification of 299 genes that were uniquely upregulated in the acute M1 and M2 stages. Amongst the most upregulated ones were multiple pro-inflammatory molecules such as interleukin 6 (IL6) and C-X-C motif chemokine ligand 5 (CXCL5). Also, matrix modifying molecules such as serine protease 35 (PRSS35) and matrix metalloproteinase (MMP1) were elevated in acute stages. Amongst the 311 genes significantly downregulated (Padj < 0.05) in the acute stages, were protease inhibitors such as serine peptidase inhibitor Kazal type 6 (SPINK6), serine protease inhibitors (SERPIN) and pro-inflammatory genes like cytochrome P450 family 1 subfamily A member 1 (CYP1A1) and aquaporin 9 (AQP9). The 10 most up- and downregulated genes exclusively in the dataset of the chronic M3 and M4 stages included genes like secreted LY6/PLAUR domain containing 1 (SLURP1) and aquaporin 5 (AQP5) which have shown to be responsible for the proliferation and differentiation of keratinocytes. Furthermore, in the chronic lesions, anti-inflammatory components such as C4b-binding protein (C4BPA), proteoglycan 4 (PRG4) and fibrinogen like 1 (FGL1) were predominantly downregulated.

Comparison of the DEGs of the M4.1 stage with the DEGs of the acute and chronic stages showed that the highest overlap was observed with the acute M1 and M2 stages with 478 DEGs versus 186 DEGs with the chronic M3 and M4 stages (Fig. 3b). Amongst the most upregulated genes were the pro-inflammatory molecules IL6 and CXCL5 and the matrix modifying MMP1 (Supplementary file 5). On the other hand, keratin 73, serpin B7 and matrix modifying inhibitor TIMP4 were amongst the most downregulated molecules in both the acute and M4.1 stages (n = 241). The most upregulated genes unique to the M4.1 stage (n = 246) included TREM1, CXCR1 and NOS2, all of which predominantly possess pro-inflammatory capacities (Supplementary file 5). The function of the most downregulated genes uniquely belonging to the M4.1 stage (n = 368) are mostly unknown (Supplementary file 5), apart from the keratinocyte-associated molecules TRPV6 and loricrin.

### qPCR analysis

The relative gene expression of IL8, SKALP and A1ML1 in almost every diseased M-stage sample was significantly higher expressed in comparison to the healthy M0 samples (Pval < 0.05). This is visualized in bar charts in supplementary file 7. The exceptions to the latter were the SKALP expression in M4 (Pval = 0.056) and M4.1 (Pval = 0.063) samples, and the IL8 expression in M4 samples (Pval = 0.056). The results of the qPCR analysis approximately confirmed the findings obtained with RNA sequencing.

## Discussion

The aim of the current study was to analyse the transcriptomic changes that occur in digital dermatitis lesions during their development and progression from healthy skin to chronic lesions and the subsequent reactivation. Principal component analysis showed a clear division between healthy tissue and the different digital dermatitis M-stages, with a further clustering of the acute M1, M2 and M4.1 stages as opposed to the chronic M3 and M4 stages. The transcriptomic analysis indicated that most of the differentially expressed genes detected in the different M stages were identical, with the upregulation of a large number of pro-inflammatory molecules and antimicrobial peptides and the downregulation of certain keratins and keratin-associated proteins. This indicates that despite the clinically healed appearance of the M3 and M4 lesions, inflammatory and anti-bacterial processes are still active in these lesions.

Interestingly, pathway analysis indicated the activation of the pro-inflammatory IL-17 signaling pathway through the upregulation of IL-17F in both acute and chronic lesions. IL-17F belongs to the IL-17 cytokine family which has shown to play a central role in the control of infections but, on the other hand, can also contribute to the pathology of numerous autoimmune and chronic inflammatory conditions^21,22^.

IL-17F can be expressed by both Th-17 cells and a subset of γδ T cells^23^. Studies show that IL-17A and IL-17F are heavily involved in lesion development in psoriasis patients, as it is increased in both serum and lesion biopsies^24^. In mice, overexpression of IL-17F is associated with neutrophilia, induction of cytokines and hyperreactivity in the airways^25^. In humans and mice, innate immune cells produce IL-17 in the skin as a way to quickly respond to injuries^26,27^. IL-17 subsequently induces the production of chemo-attractants for lymphocytes, monocytes, neutrophils and dendritic cells^28^. Studies have shown that the levels of IL-8 and IL-6 produced by keratinocytes were significantly higher following stimulation with IL-17F in comparison to other stimulants such as TNF-alpha or IL-17A^29,30^. IL-8 and IL-6 were amongst the 10 highest upregulated genes in the M1 and M2 lesions, suggesting that IL-17F potentially plays a role as a chemokine producing stimulant and neutrophil stimulant in digital dermatitis affected skin. The involvement of the IL-17 pathway in digital dermatitis lesions has not been described before and therefore forms an interesting area for future research. In a study by Newbrook et al. (2021), ingenuity path analysis (IPA) predicted the enrichment of several pathways such as the TREM1- and IL-17 signaling pathways in fibroblasts after being challenged with treponemes^31^.

Alpha2-macroglobulin-like protein 1 (A2ML1) was one of the most upregulated genes in all the different disease stages. This is in agreement with earlier findings of Scholey et al., in which a significant A2ML1 upregulation was found in M2 lesion biopsies^9^. This molecule possibly plays an important role during keratinocyte desquamation by inhibiting extracellular proteases^32^. Previous research has shown that A2ML1 plays a beneficial role in the growth and the survival of syphilitic and periodontitis-associated treponemes^9,33,34^. It is plausible that DD-associated treponemes operate in a similar way, since treponemes have been found in any type of M-stage^7^. Another gene that appears in the top three overall most upregulated genes, is the skin protective gene coding for elafin (PI3). Concomitantly, an elafin-like gene is upregulated in all M-stages of the dataset. Elafin, also called SKALP, is a skin-derived inhibitor of polymorphonuclear leukocyte elastase and proteinase, and also enables cross-linking to extracellular matrix proteins^35^. Research on psoriasis has previously suggested that elafin protects the skin against damage generated by the influx of leukocytes^36^. Therefore, the rise in elafin and elafin-like protein levels in DD affected tissue might be an attempt of the host to protect the tissues against the damage associated with the high influx of neutrophils, eosinophils, plasma cells and monocytes^4,8^. Antimicrobial peptides LAP and TAP belong to the highly conserved β-defensin subfamily and serve as the first line of host defense. A study evaluating the effect of human β-defensins on the life span of neutrophils, indicated that these antimicrobial peptides are able to suppress neutrophil apoptosis through the CC chemokine receptor 6^37^. The β-defensins directly kill bacteria, and thus possibly have an effect on the survival of treponemes. Molecules such as secretoglobin (SCGB1D) and odorant binding protein-like molecule (MGC151921) that have a negative effect on inflammation and chemotaxis appear to be heavily suppressed. An abundance of keratins and keratin-associated proteins were furthermore downregulated in all the different M-stages. The latter are known to be crucial components of the skin barrier and integrity. Contrastingly, the keratins KRT6 and KRT17 that function as post-injury 'alarmins' were significantly upregulated in all M-stages (Padj < 0.05). Physiologically, these keratins stimulate keratinocytes during wound repair until the skin barrier is repaired^38^. Their consistent upregulation suggests in the direction of the establishment of a chronic wound due to the failure to restore the superficial skin barrier.

Besides the set of genes that are differentially expressed throughout all the M-stages, some pro-inflammatory proteins seemed to be exclusively upregulated in the acute stages, including the pro-inflammatory cytokines IL-6 and CXCL5. IL-6 is a major cytokine that promotes the early stages of inflammation and wound healing. A dysregulation of IL-6 can be found in psoriatic skin or impaired wound repair, and can potentially exacerbate the concomitant epidermal hyperplasia^39,40^. CXCL5 on the other hand has been implicated in the homeostasis and chemotaxis of neutrophils. Moreover, the upregulation of these pro-inflammatory compounds in the acute lesions coincides with the significant downregulation (Padj < 0.05) of the anti-inflammatory molecule SPINK6, which acts in the epidermis through its inhibitory activity on kallikrein 5 and 14^41,42^. Immunohistochemical staining has also indicated a reduced SPINK6 expression in atopic dermatitis and psoriasis lesions^41^. Importantly, IL6 and CXCL5 were no longer significantly upregulated in the M3 and M4 lesions, neither was the anti-inflammatory SPINK6 downregulated (Padj < 0.05). In the M4.1 stage on the other hand, which is considered to be the resurgence of an M4 to an acute M1 lesion, the expression of CXCL5 and IL6 was switched back on again, even at a higher level compared to the acute M1 and M2 stages. These results indicate that the partial healing observed in the chronic stages is associated with a temporary dampening of these pro-inflammatory molecules.

The processes involved in the healing that is typically observed in the M4 lesions need, however, further clarification. The expression of genes vital to wound healing, such as HIF1A and matrix metalloproteinases (e.g. MMP9) were apparently not impacted^43^. Yet several fibroblast growth factor-coding genes crucial for post-traumatic skin regeneration appear to be even downregulated. The latter combined with a downregulation of several metalloproteinase inhibitor (TIMP) genes, can lead to a disproportionate enzymatic breakdown of the extracellular matrix. Interestingly however, TIMP4 seems to be downregulated only in the acute stages M1, M2 and the M4.1-stage, whereas TIMP2 in the chronic stage M4 and TIMP3 in the M3 stage. These findings point towards a partial activation of wound repair mechanisms with a specific imbalance of the extracellular matrix-associated repair.

In conclusion, the present study presents a global overview of the host response associated with all clinical stages of digital dermatitis. The findings indicate a massive upregulation of a wide array of pro-inflammatory molecules, antimicrobial peptides and other skin-protective genes whereas several anti-inflammatory proteins, keratins and components of the skin barrier are downregulated. The combination of this implies a substantial disruption of the integrity of the skin barrier concomitant with a chronic inflammation. Most innovative findings of this study were the activation of the pro-inflammatory IL-17 signaling pathway in all the M stages, through the upregulation of IL-17F, and the fact that an inflammatory response is still measurable inside the M4 lesions, despite their healed appearance. In addition, our results show a strong resurgence of pro-inflammatory cytokines in the M4.1 stage, indicating the flare-up of an acute lesion. This acute flare-up can easily be missed in this overall healed appearing stage during routine claw trimming, and thus escape from treatment and subsequently remain as an infectious reservoir within the herd. An early recognition in combination with thorough treatment of these lesions might prevent a resurgence of acute lesions in the herd, with its accompanying economical and animal welfare consequences. Regardless of the inflammatory hostile environment, treponemes seem to thrive well in the typical lesions. Therefore, more in-depth research on the interaction between host molecules and treponemes is essential to understand how these bacteria are able to evade this immune attack and at the same time continuously trigger an inflammatory response. Furthermore, the identification of IL-17F as a potential key regulator opens perspectives to investigate potential anti-inflammatory treatment strategies.

## Supplementary Information

Supplementary Information 1.

Supplementary Information 2.

Supplementary Information 3.

Supplementary Information 4.

Supplementary Information 5.

Supplementary Information 6.

Supplementary Information 7.

## Data Availability

The sequencing data reported in the present paper can be found in the supplementary files.
