# Supplementary material for: Continuous activation of the IL-17F driven inflammatory pathway in acute and chronic digital dermatitis lesions in dairy cattle
Source: Sci Rep. 2022 Aug 18;12:14070. doi: 10.1038/s41598-022-17111-4 (PMC9388621; doi:10.1038/s41598-022-17111-4)
Supplement: Supplementary file 6 — Supplementary Information 6. [file 41598_2022_17111_MOESM6_ESM.pdf]

**Supplementary file 6: The primer sequence, accession number and source of the primers used for the subset of 5 genes utilized in qPCR analysis of the given samples.**

| Gene | Primer sequence (5'-3') | GenBank Accession number | Source |
|---|---|---|---|
| **GADPH** | F: GGGTCATCATCTCTGCACCT<br>R: GGTCATAAGTCCCTCCACGA | NM_001034034.1 | Refaai et al., 2013 |
| **RPL0** | F: CTTCATTGTGGGAGCAGACA<br>R: GGCAACAGTTTCTCCAGAGC | NM_001012682.1 | Refaai et al., 2013 |
| **IL-8** | F: GTTGCTCTCTTGGCAGCTTT<br>R: GGTGGAAAGGTGTGGAATGT | NM_173925.2 | Refaai et al., 2003 |
| **A2ML1** | F: GCCTCGCTGCCTTCAATCTA<br>R: GCCACATTCCAAGATGCCAC | NM_001191301.1 | NCBI database |
| **SKALP** | F: GTCCACTCCTCCCTAAGCGT<br>R: AGAGGGTCATGCTACAGGCA | XM_005214890.4 | NCBI database |
