# Supplementary material for: Continuous activation of the IL-17F driven inflammatory pathway in acute and chronic digital dermatitis lesions in dairy cattle
Source: Sci Rep. 2022 Aug 18;12:14070. doi: 10.1038/s41598-022-17111-4 (PMC9388621; doi:10.1038/s41598-022-17111-4)
Supplement: Supplementary file 7 — Supplementary Information 7. [file 41598_2022_17111_MOESM7_ESM.pdf]

Supplementary file 7: A display of the relative gene expression levels in the different DD stages for A2ML1, SKALP and IL8. The data was obtained through qPCR analysis. Each bar represents the mean value of a sample group. The standard error is shown layered on top of the bars. The asterisk above a bar indicates there's a significant difference between the relative gene expression from the healthy M0-stage and the indicated M-stage. The threshold of significance is set at Pval <0.05.

(**a**) The expression of A2ML1 in the diseased DD samples is significantly different from the healthy M0 samples. The P-value for the M1, M2, M3 and M4 data (0.008) is <0.01 and <0.05 for M4.1 (0.016).

(**b**) The expression of SKALP in the M1, M2 and M3 samples is significantly different from the healthy M0 samples. The p-values for M4 (0.056) and M4.1 (0.063) are close to 0.05 but are not significant.

(**c**) The expression of IL8 in the M1, M2, M3 and M4.1 samples is significantly different from the healthy M0 samples. The p-value for M4 (0.056) approximates the threshold of 0.05 but is not significant.

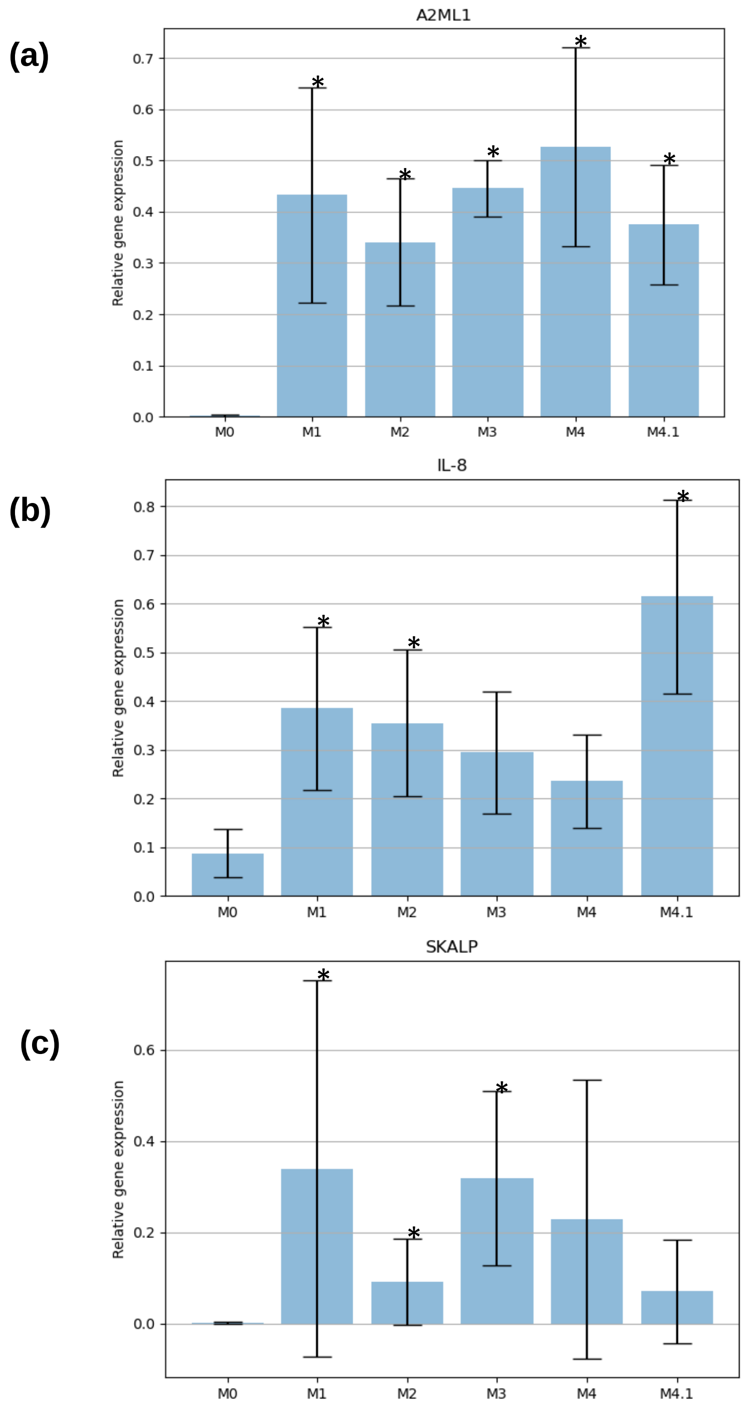
(a)
A2ML1
Relative gene expression
0.0
0.1
0.2
0.3
0.4
0.5
0.6
0.7
M0
M1
M2
M3
M4
M4.1
(b)
IL-8
Relative gene expression
0.0
0.1
0.2
0.3
0.4
0.5
0.6
0.7
0.8
M0
M1
M2
M3
M4
M4.1
(c)
SKALP
Relative gene expression
0.0
0.2
0.4
0.6
M0
M1
M2
M3
M4
M4.1
